# General attachment and behavioral problem among Chinese adolescents: a serial multiple mediation model

**DOI:** 10.3389/fpsyg.2025.1621111

**Published:** 2025-08-13

**Authors:** Yuting Cao, Hao Zhang, Xiancai Cao, Dahua Wang

**Affiliations:** ^1^Faculty of Psychology, Tianjin Normal University, Tianjin, China; ^2^Key Research Base of Humanities and Social Sciences of the Ministry of Education, Academy of Psychology and Behavior, Tianjin Normal University, Tianjin, China; ^3^Tianjin Social Science Laboratory of Students’ Mental Development and Learning, Tianjin, China; ^4^Institute of Developmental Psychology, Beijing Normal University, Beijing, China

**Keywords:** general attachment, parent–child attachment, self-control, dual-system model, behavioral problems

## Abstract

**Introduction:**

Grounded in the attachment hierarchy model and attachment control system model, this study explored the link between general attachment and adolescent behavioral problems, focusing on the sequential mediation of parent–child attachment and self-regulation within the dual-system framework.

**Methods:**

A survey of 568 adolescents (M = 16.58 years) assessed general and parent–child attachment, self-control, and behavioral problems.

**Results:**

Results showed that general attachment predicted behavioral problems via parent–child attachment, which was negatively associated with general attachment. Moreover, attachment avoidance predicted behavioral problems through the control system, while attachment anxiety did so via the impulsive system.

**Discussion:**

These findings highlight distinct pathways linking attachment dimensions to adolescent behavior, supporting both theoretical models.

## Introduction

During adolescence, individuals undergo rapid physiological and psychological transformations, including hormonal fluctuations, physical growth, heightened emotional reactivity, and advancing cognitive abilities (Mastorci et al., 2024; Özdemir et al., 2016; Uktamovna, 2025). However, psychological and behavioral development often lags behind physical maturation (Jimenez et al., 2023). In this stage, adolescents strive for psychological independence while navigating the tension between maintaining emotional attachment to parents and seeking acceptance and belonging within peer groups—a dynamic that is frequently associated with the emergence of internalizing and externalizing behavioral problems (Badenes-Ribera et al., 2019; Rejaän et al., 2022). Adolescent behavioral problems refer to the physical and psychological impairments that arise in family, school, and society when an individual’s behavior cannot adapt to changing environments (Achenbach et al., 1991). Research has shown that adolescent problem behaviors are closely related to emotional and anxiety disorders, as well as suicidal tendencies (Danielsen et al., 2025; Sivertsen et al., 2024). These behavioral patterns may not only contribute to academic struggles but also impair peer interactions (Sentse et al., 2017). Moreover, these patterns of behavior exhibit strong links to adverse consequences, including substance dependency and illegal conduct (De Geronimo et al., 2024; Rowland et al., 2021; Saladino et al., 2021). Thus, understanding the underlying factors contributing to adolescent behavioral issues and implementing timely interventions remains crucial.

### Parent–child attachment and behavioral problems

According to family systems theory, the family is a crucial environment for adolescent development and serves as an important microsystem influencing their growth (Cox and Paley, 2003). As a key component of the family system, parent–child attachment plays a significant role in shaping individuals’ social behavior development. In caregiving, attachment between parents and children is referred to as parent–child attachment (Armsden and Greenberg, 1987; Bowlby, 1969; Yin et al., 2021). Individuals’ overall well-being, both physiological and psychological, is deeply influenced by this attachment (Risi et al., 2021; Tan et al., 2023). Although the targets of attachment become increasingly diverse during adolescence, with peers (Delgado et al., 2022) and teachers (Fabris et al., 2022) also emerging as important attachment figures, the role of parent–child attachment remains critical (Gorrese and Ruggieri, 2012; Wang et al., 2025). Research suggests that the emotional connection between adolescents and their parents can serve as either a foundation for resilience or a contributing factor to the emergence of behavioral difficulties (Madigan et al., 2016; Sesti Becker et al., 2019; Fuentes-Balderrama et al., 2023). According to studies, adolescents who lack a secure relationship with their parents may be at higher risk for emotional and behavioral difficulties (Fuentes-Balderrama et al., 2023). These may manifest as feelings of loneliness and depression (Yildiz, 2016) or disordered eating patterns like emotional anorexia (Laporta-Herrero et al., 2021). In contrast, a secure and nurturing relationship between parents and children contributes to improved self-efficacy and psychological resilience (Chen et al., 2019; Spruit et al., 2020; Tan et al., 2023); adolescent conflict is reduced, parental intimacy is increased, friendship quality improves, and problem behaviors are decreased (Chevalier et al., 2023; Khan et al., 2020; Zimmermann and Iwanski, 2019).

### General attachment and behavioral problems

In addition to the attachment model specific to relationships, such as parent–child attachment, general attachment significantly predicts adolescent psychological and behavioral problems (Lee and Hankin, 2009; Zhang X. et al., 2022; Zhang Z. et al., 2022). Bowlby (1969) proposed that through ongoing interactions with attachment figures, individuals gradually develop internal working models—mental representations of the self and others in relationships. This process leads individuals to develop relatively stable mental representations of the self and others, thereby forming a general attachment orientation. General attachment is typically conceptualized along two dimensions: attachment anxiety, characterized by a heightened fear of rejection or abandonment in close relationships, and attachment avoidance, marked by discomfort with closeness and intimacy due to fear of dependence and lack of trust (Bowlby, 1969). A secure attachment relationship serves two core functions (Ainsworth and Bowlby, 1991; Hazan and Shaver, 1994). First is the “safe haven” function, in which the attachment figure provides emotional comfort and a sense of security in times of stress or threat. Second is the “secure base” function, whereby the presence of a trusted attachment figure supports the individual’s exploration of the environment and engagement in social interactions. As Bowlby argued, attachment is an essential mechanism for dealing with external threats and stresses, providing emotional security and psychological support, and coping with uncertainty. Previous studies have found a connection between both attachment anxiety and avoidance with emotional and behavioral issues in adolescents. Lee and Hankin (2009) and Zhang X. et al. (2022) showed that individuals with elevated attachment anxiety and avoidance are at a higher risk for experiencing depression and behavioral issues. In contrast, secure attachment promotes emotional regulation and increases prosocial behavior in adolescents (Costa Martins et al., 2022; Domic-Siede et al., 2024; Singh and Chauhan, 2025). Groh et al. (2017) conducted a meta-analysis showing that securely attached children tend to exhibit fewer externalizing and internalizing behaviors compared to those with insecure attachments. In summary, adolescents’ psychological and behavioral issues are associated with general attachment.

### General attachment, parent–child attachment, and behavioral problems

Existing research has primarily focused on how relationship-specific attachments (Paquette et al., 2024; Valarezo-Bravo et al., 2024; Wang J. et al., 2021; Wang Y. et al., 2021) or general attachment representations predict adolescent behavioral problems (Blake et al., 2024; Wambua et al., 2018), with less attention given to the hierarchical structure of the attachment system. According to Collins and Read’s (1994) attachment hierarchy model, general attachment representations occupy the highest level, characterized by a high degree of generalization, while relationship-specific attachments are situated at lower levels. Both levels influence an individual’s psychological and behavioral outcomes (Mikulincer and Shaver, 2010). Fraley (2002) prototype theory further suggests that general attachment representations established in early childhood continue to influence behaviors across all types of interpersonal relationships. Moreover, the emotional security hypothesis, proposed by Davies and Cummings (1994), suggests that insecure parent–child attachments disrupt the development of secure bonds, which in turn leads to emotional insecurity in adolescents. However, research on how different levels of attachment representations jointly predict adolescent problem behaviors remains relatively limited (Cao et al., 2024). Our study hypothesizes that general attachment predicts adolescent problem behavior through parent–child attachment.

### Self-control and problem behaviors

Self-control involves an individual’s capacity to manage their thoughts, emotions, and actions in accordance with personal or societal standards, balancing immediate impulses with future goals (Baumeister et al., 2007). This ability plays a key role in influencing adolescent behavioral issues. The dual-system theory points out that self-control consists of two primary components: the impulse system, which responds quickly to emotions, external stimuli, and rewards, driving immediate gratification; and the control system, which plays a more advanced role by inhibiting impulsive behaviors, facilitating thoughtful decision-making, and regulating emotions (Hofmann et al., 2009). During adolescence, these two systems often develop unevenly: the impulse system is hyperactive, making adolescents highly attracted to pleasure and rewards, while the control system is underdeveloped, leading to difficulties in behavior regulation. This imbalance raises the likelihood of engaging in behaviors such as substance abuse, pathological gambling, and internet addiction (Somerville et al., 2010; Steinberg, 2010). Considering the functional differences between the impulse and control systems and their developmental imbalance during adolescence, research from a dual-system perspective can offer valuable insights into how self-control predicts adolescent problem behaviors. However, existing studies have mostly treated self-control as a unitary construct, with relatively few distinguishing between the two systems when examining their roles in adolescent behavioral problems.

### General attachment, self-control, and problem behaviors

Adolescence is a critical developmental stage during which individuals transition from dependence on the family to relative independence (Allen and Hauser, 1996). During this period, the attachment relationships formed within the family play a vital role in the development of self-control. General attachment may predict problem behaviors indirectly through self-control. Individuals with insecure attachment often experience persistent negative emotions, such as fear of abandonment or distrust of others. Regulating these emotions consumes substantial cognitive resources, thereby impairing executive control functions and lowering self-control capacity (Gifford, 2002). Empirical studies support this view. Tangney et al. (2018) found that self-control is strongly linked to attachment styles. After adjusting for social desirability, a secure attachment style was positively associated with self-control, while both avoidant and anxious styles showed a negative correlation. Research also shows that self-control deficits have been linked to various psychological issues such as anxiety, depression, aggression, and addiction (Blase et al., 2021; Chen et al., 2019; Morshedi and Mohamadi, 2024; Pan et al., 2024; Zhang X. et al., 2022; Zhang Z. et al., 2022). Accordingly, this study hypothesized that self-control could mediate the relationship between general attachment and problematic behaviors. This mechanism is particularly critical during adolescence. Because adolescents have heightened emotional reactivity and underdeveloped self-regulation systems (Somerville et al., 2010; Steinberg, 2010), they are especially vulnerable to the disruptive effects of insecure attachment on self-control, which in turn increases the risk of problem behaviors. Therefore, self-control may represent a key pathway linking general attachment styles to adolescent problem behaviors.

However, attachment anxiety and attachment avoidance may predict behavioral problems through different self-control systems. According to the attachment control system model, when proximity-seeking fails, individuals may turn to secondary strategies such as hyperactivation or deactivation. For those with attachment anxiety, the need for attachment figures increases, often leading to heightened impulsivity (Mikulincer and Shaver, 2010). Due to the underdeveloped cognitive control system, adolescents struggle to regulate risky impulses, which increases their likelihood of engaging in externalizing behaviors such as impulsivity and emotional dysregulation (Estévez et al., 2018). Attachment avoidants, however, tend to use deactivation strategies to maintain emotional distance by suppressing negative emotions and thoughts (Mikulincer and Shaver, 2010). However, this suppression process depletes cognitive resources, reducing the resources available to the control system, thus weakening self-regulation (Baumeister and Vohs, 2007) and ultimately increasing the risk of problem behaviors. Individuals with attachment anxiety are often more impulsive, which increases the likelihood of exhibiting externalizing problem behaviors. In contrast, attachment avoidance is linked to diminished self-regulation, resulting in reduced self-control and a heightened risk of problem behaviors.

### Parent–child attachment, self-control, and problem behaviors

In addition, adolescent problem behaviors may also be predicted by parent–child attachment and self-control. Self-control mediates the link between parent–child attachment and behavioral problems (Sun et al., 2022). In the family environment, parent–child attachment forms the foundation for parental nurturing, enabling effective monitoring, identification of deviant behaviors, and timely correction, thus teaching children to control impulses and consider long-term consequences (Farley and Kim-Spoon, 2014; Li et al., 2019). Adolescent self-control has been linked to high-quality parent–child attachments (Li et al., 2019; Sun et al., 2022). It has been shown that adolescents who maintain strong self-control have fewer aggressions and rule-breaking behaviors and fewer academic and psychological difficulties (Van der Ende et al., 2016). Therefore, adolescent problem behaviors are mediated by self-control in parent–child attachments.

In summary, adolescents’ psychological development is profoundly influenced by the family ecological system. The family is not only the starting point for the formation of attachment relationships but also a critical environment for the development of self-control. A warm and supportive family environment facilitates the development of secure attachment and strong self-control, thereby reducing the likelihood of problem behaviors (Bronfenbrenner, 1979).

However, several gaps remain in the existing research. First, as adolescents’ attachment figures become increasingly diverse, the structure of attachment representations also grows more complex. Examining how different levels of attachment representations (i.e., general attachment and parent–child attachment) are associated with problem behaviors may offer deeper insights into the potential mechanisms underlying the attachment system’s links with adolescent behavioral development. To our knowledge, only one study to date has simultaneously examined the effects of both general and parent–child attachment on adolescent behavioral problems (Cao et al., 2024). Second, according to the dual-systems theory of self-control, the impulse and control systems develop asynchronously during adolescence: adolescents typically display stronger impulsivity while their control system remains immature (Steinberg, 2010). Moreover, different types of insecure attachment may be differentially associated with problem behaviors via distinct self-control pathways: attachment anxiety may predict problem behaviors through the impulse system, while attachment avoidance may do so through the control system. Adopting a dual-systems perspective allows for a clearer understanding of the mechanisms linking attachment and problem behaviors via self-control. However, most previous studies have treated self-control as a unitary construct, with few distinguishing the functions of its two systems when exploring its relationship with adolescent problem behaviors.

**Figure 1:**
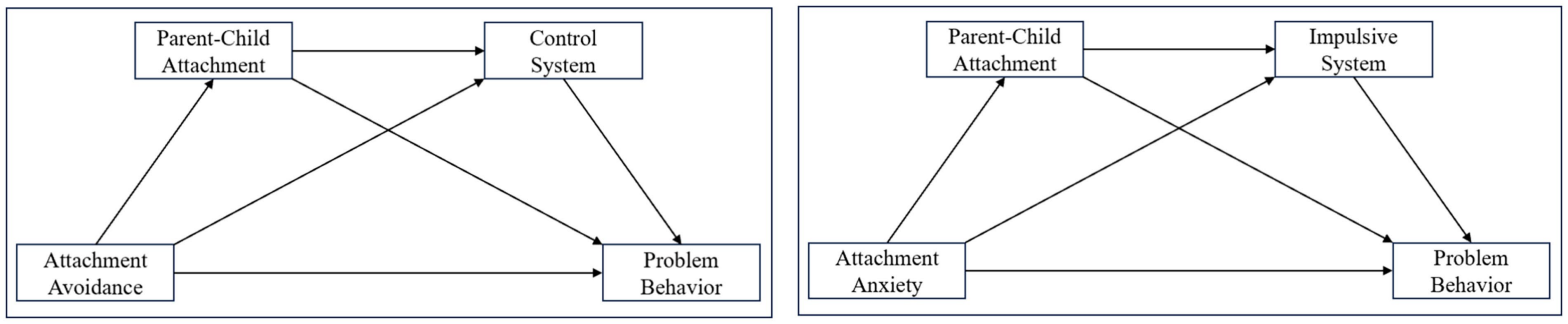
Hypothesized model of the study.

Based on this, the present study adopts a family ecological framework and integrates the attachment hierarchy model with the dual-systems theory of self-control to construct a chain mediation model. This model aims to systematically examine the roles of general attachment, parent–child attachment, and the two self-control systems in predicting adolescent problem behaviors (Figure 1). Our hypotheses are as follows:

*Hypothesis 1*: Parent–child attachment mediates the effect of general attachment on adolescent behavioral problems.

*Hypothesis 2*: Attachment anxiety and avoidance predict behavioral problems through different routines; attachment anxiety predicts problem behaviors through the mediation of the impulse system, while attachment avoidance predicts problem behaviors through the mediation of the control system.

*Hypothesis 3*: The dual-systems model of self-control acts as a mediator in the relationship between general attachment and behavioral problems.

## Method

### Participants

This study adopted a convenience sampling method. Researchers contacted schools in their home provinces (Hebei, Jiangsu, Henan, Guangxi, and Xinjiang), and one middle or high school was selected from each province. A total of 568 questionnaires were distributed across five schools, and 434 valid responses were collected, yielding a response rate of 76.40%. Participants ranged in age from 13 to 20 years (mean age = 16.58, *SD* = 1.50). Among them, 233 were male (53.7%) and 201 were female (46.3%). The sample included students from multiple grade levels: 46 seventh-grade students, 76 ninth-grade students, 283 eleventh-grade students, and 29 twelfth-grade students.

### Procedures

This study employed a cross-sectional survey design, and the data collected was from a larger study on adolescent attachment. After coordination between the research team and participating schools, data were collected at the class level by administering and collecting questionnaires uniformly within the classroom setting. The survey was conducted during a regular 45-min class period, supervised and guided by trained research assistants. At the beginning of each session, research assistants explained the study’s purpose, provided instructions for completing the questionnaire, and highlighted relevant precautions. Participants were encouraged to ask questions freely to ensure full understanding. All participants took part voluntarily after informed consent was obtained from the schools, class teachers, parents, and the students themselves. The study protocol was approved by the Ethics Committee of the Faculty of Psychology at Tianjin Normal University. To express appreciation, small gifts (e.g., notebooks, pens, candies) were provided to each participant during data collection.

### Measures

#### Experiences in close relationships–relationship structures (ECR-RS)

The Experiences in Close Relationships–Relationship Structures (ECR-RS) questionnaire measured general attachment relationships across different life stages (Fraley et al., 2011). A total of nine items comprise the scale, which evaluates two dimensions: anxiety and avoidance. A study conducted by Feddern Donbaek and Elklit (2014) has shown that the scale is reliable and valid in adolescent populations. In this study, Cronbach’s α for attachment avoidance and anxiety were 0.71 and 0.82, respectively.

#### Inventory of parent and peer attachment (IPPA)

The Inventory of Parent and Peer Attachment (IPPA), originally developed by Armsden and Greenberg (1987), was utilized to evaluate parent–child attachment. This study employed the mother and father subscales, each consisting of 15 items that assess trust, communication, and alienation (reverse-scored). Parent–child attachment was measured on a 5-point scale, with the overall score derived from the combination of trust, communication, and alienation dimensions. Analysis indicated good internal consistency across the three subscales (Guarnieri et al., 2010). In this study, Cronbach’s α values for father-child and mother–child attachment were 0.75 and 0.73, respectively.

#### Dual-mode of self-control scale (DMSC)

The Dual-Mode of Self-Control Scale (DMSC), adapted for use with Chinese middle school students, was designed to assess self-regulation capacities (Xie et al., 2014). This measure comprises 21 items categorized into two broad components. The impulsivity-related dimension includes aspects such as heightened impulsivity, proneness to distraction, and difficulty in delaying gratification. In contrast, the regulatory dimension encompasses problem-solving skills and a future-oriented perspective. Responses are recorded on a five-point Likert-type scale, with scores derived by averaging the relevant item responses. Higher scores reflect stronger tendencies toward either impulsivity or self-regulation. Prior research has demonstrated its robust psychometric properties for evaluating self-control in Chinese adolescents. In the present study, the Cronbach’s α values for impulsivity and self-regulation subscales were 0.86 and 0.84, respectively.

#### Strengths and difficulties questionnaire (SDQ)

The student-adapted version of the Strengths and Difficulties Questionnaire (SDQ), revised by Kou et al. (2007), was employed to evaluate behavioral tendencies and social adjustment in students over the past 6 months. This instrument consists of 25 items, classified into four domains addressing difficulties: emotional distress, behavioral regulation issues, attentional deficits/hyperactivity, and peer interaction challenges. Additionally, one dimension assesses prosocial behavior. Responses are rated on a three-point Likert scale, and a composite difficulty score is calculated based on the sum of the four problem-related subscales. Prior research has established the SDQ as a psychometrically sound measure for assessing emotional and behavioral characteristics in adolescents. In this study, the Cronbach’s α for the difficulty subscale was 0.73.

### Statistical analysis

Descriptive statistics were performed to summarize the dataset, while Pearson correlations were conducted to investigate the associations among the key variables. Mediation analyses were implemented through the PROCESS macro in SPSS (Hayes, 2013). A bias-corrected bootstrapping approach with 10,000 resamples was used to estimate indirect effects, generating 95% confidence intervals to evaluate their statistical significance. Statistical significance is determined by not including zero in the BCBI distribution (Preacher and Hayes, 2008). Four analyses were conducted using PROCESS to estimate each mediation model. The direct effect refers to the estimated association between the two general attachment styles (avoidance and anxiety) and adolescent problem behaviors. The indirect effect is the estimate of how general attachment predicts problem behaviors through (a) parent–child attachment, (b) the control or impulsive systems, or (c) parent–child attachment and the control or impulsive systems. Adolescent problem behaviors show associations with both direct and indirect pathways through attachment. The study accounted for key sociodemographic factors, including age, gender, and economic background, to ensure robust mediation analysis. All statistical procedures were carried out using IBM SPSS 26.0.

Further, we performed a post-hoc power analysis using the Monte Carlo Power Analysis for Indirect Effects simulation by Schoemann et al. (2017). We utilized this power analysis to produce effect sizes for indirect effects using correlation values. The smallest power of the indirect path in our model was 0.6.

## Results

### Descriptive statistics and associations

Table 1 presents the means, standard deviations, and correlations among the study variables. The mean self-reported problem behavior score among adolescents was 13.53 (*SD* = 5.19). Significant correlations were observed among attachment avoidance, parent–child attachment, self-regulation, and behavioral problems. Notably, elevated attachment anxiety corresponded to reduced behavioral problems, whereas attachment avoidance was linked to a heightened occurrence of such behaviors.

**Table 1:** Descriptive statistics and variable associations (*n* = 434).

Variable	*M*	*SD*	1	2	3	4	5	6	7	8	9
1. Gender	0.46	0.50	1								
2. Age	16.58	1.50	−0.02	1							
3. Family income	3.55	1.47	−0.05	0.19**	1						
4. Parent–child attachment	38.05	18.67	−0.06	0.01	0.13**	1					
5. Attachment anxiety	4.71	1.48	0.24**	−0.03	0.00	−0.14**	1				
6. Attachment avoidance	3.69	0.99	−0.03	−0.08	−0.08	−0.33**	0.07	1			
7. Impulsive system	7.94	1.81	0.06	0.09	−0.02	−0.30**	0.31**	0.15**	1		
8. Control system	6.85	1.10	−0.09	0.05	0.09	0.37**	−0.06	−0.24**	−0.25**	1	
9. Problem behaviors	13.53	5.19	0.11*	−0.02	−0.08	−0.35**	0.41**	0.33**	0.54**	−0.27**	1

Gender is coded as a dummy variable (female = 1, male = 0), with the mean indicating the proportion of female participants.

*p < 0.05, **p < 0.01.

### Sequential mediation of parent–child attachment and self-control

**Figure 2:**
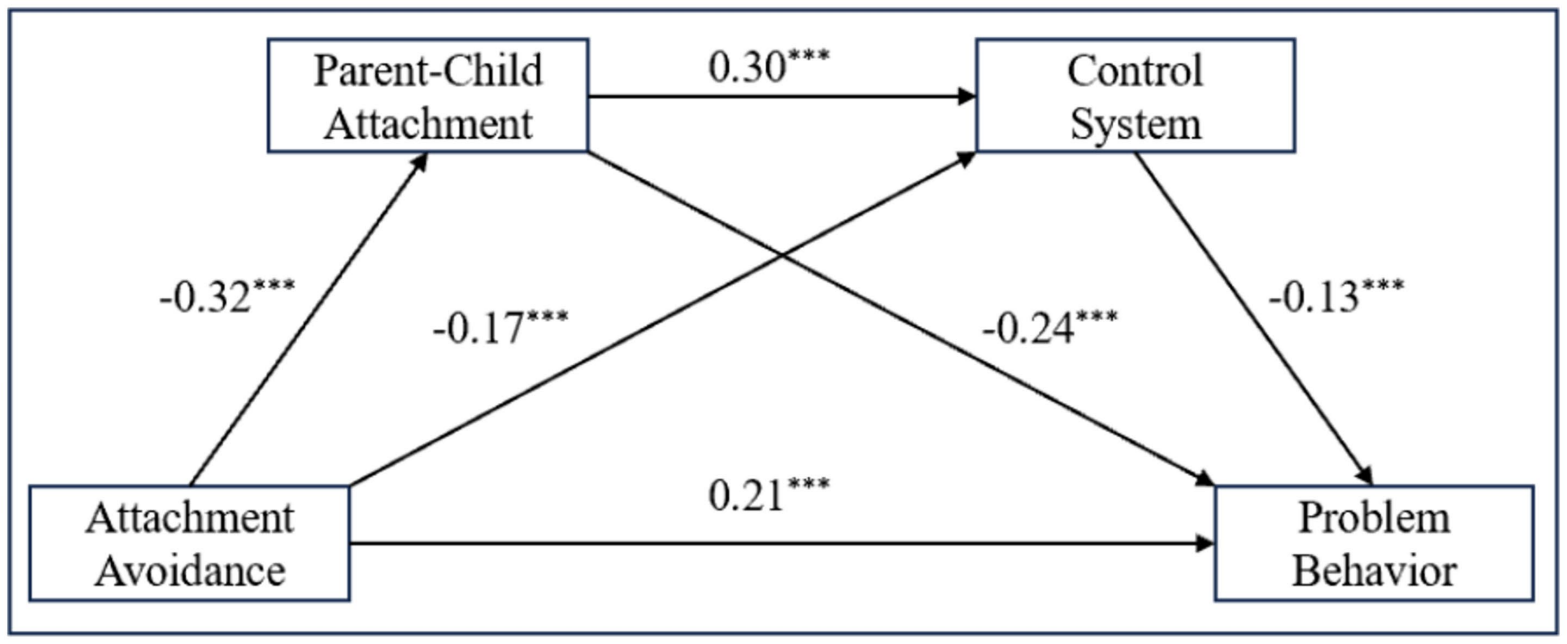
Standardized regression weights for attachment avoidance, the control system, and problem behaviors. *p < 0.05, **p < 0.01, ***p < 0.001.

The coefficients presented in Figure 2 indicate that attachment avoidance had a significant effect on problem behaviors (*β* = 0.21, *t* = 6.76, *p* < 0.001). Direct effects of attachment avoidance were significant on both parent–child attachment (*β* = −0.32, *t* = −6.67, *p* < 0.001) and control system (*β* = −0.17, *t* = −3.45, *p* < 0.001). Parent–child attachment served as a significant mediator in the relationship between attachment avoidance and control system (*β* = 0.30, *t* = 6.12, *p* < 0.001). Both parent–child attachment (*β* = −0.24, *t* = −4.65, *p* < 0.001) and control system (*β* = −0.13, *t* = −2.59, *p* < 0.001) significantly predicted problem behaviors.

Table 2 outlines the results from the Bootstrap analysis, highlighting significant indirect effects through parent–child attachment (*β* = 0.40, 95% CI = [0.20, 0.66]), the control system (*β* = 0.12, 95% CI = [0.01, 0.26]), and a combined indirect effect (*β* = 0.07, 95% CI = [0.01, 0.15]). The total indirect effect was found to be 0.59 (95% CI = [0.33, 0.90]), supporting the hypothesized chain mediation.

**Table 2:** Mediation effect of attachment on the impulsive system.

	Effects	Standard errors	95% Confidence interval	
			Lower	Upper
Problem behaviors				
Total effect: attachment avoidance → Problem behaviors	1.71 ***	0.25	1.21	2.21
Direct effect: attachment avoidance → Problem behaviors	1.13 ***	0.26	0.62	1.64
Indirect effects from avoidance to problem behaviors by:	0.59	0.15	0.33	0.90
Parent–Child attachment	0.40	0.12	0.20	0.66
Control system	0.12	0.06	0.01	0.26
Parent–Child attachment → Control system	0.07	0.04	0.01	0.15
Problem behaviors				
Total effect: attachment anxiety → Problem behaviors	1.56***	0.17	1.23	1.88
Direct effect: attachment anxiety → Problem behaviors	1.00***	0.15	0.71	1.30
Indirect effects from anxiety to problem behaviors by:	0.56	0.12	0.33	0.79
Parent–Child attachment	0.09	0.05	0.00	0.20
Impulsive system	0.42	0.09	0.25	0.62
Parent–Child attachment → Impulsive system	0.04	0.03	0.00	0.11

***p < 0.001.

### Serial-multiple mediation of parent–child attachment and the impulsive system

**Figure 3:**
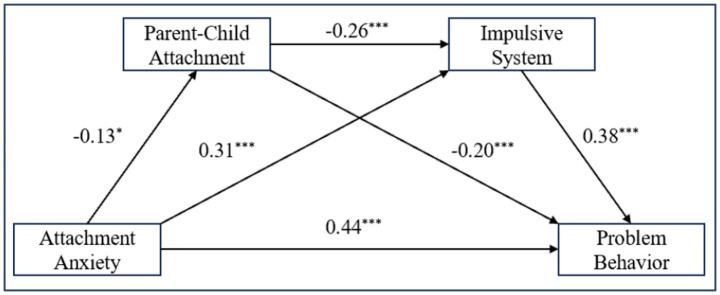
Standardized regression weights for attachment anxiety, the impulsive system, and problem behaviors. *p < 0.05, **p < 0.01, ***p < 0.001.

Figure 3 shows the findings from the second analysis, with attachment anxiety significantly predicting adolescent problem behaviors (*β* = 0.44, *t* = 9.45, *p* < 0.001). The direct effects of attachment anxiety on parent–child attachment (*β* = −0.13, *t* = −2.51, *p* < 0.05) and the impulsive system (*β* = 0.31, *t* = 6.56, *p* < 0.001) were significant. Additionally, the mediation effect of parent–child attachment on the impulsive system was significant (*β* = −0.26, *t* = −5.52, *p* < 0.001). Both parent–child attachment (*β* = −0.20, *t* = −4.91, *p* < 0.001) and the impulsive system (*β* = 0.38, *t* = 8.74, *p* < 0.001) significantly predicted adolescent problem behaviors.

In Table 2, the indirect effect through the impulsive system as a mediator was 0.42 (95% CI = [0.25, 0.62]). The indirect effect via parent–child attachment was 0.09 (95% CI = [−0.00, 0.20]), and the combined effect of both parent–child attachment and impulsive behavior was 0.04 (95% CI = [−0.00, 0.11]). However, none of these effects reached statistical significance, with the bootstrap confidence intervals close to the critical value.

## Discussion

This research merges the dual-system model of self-control with the chain mediation model of parent–child attachment to analyze how general attachment impacts adolescent problem behaviors. The results were mostly in line with our expectations, though some were not.

Adolescent problem behaviors were associated with general attachment, parent–child attachment, and dual self-control, as predicted. The findings of the correlation analysis revealed a moderate-to-low negative correlation between general and parent–child attachment, which aligns with previous research (Gillath et al., 2016; Wang et al., 2015; Wang and Wang, 2012; Cao et al., 2024). According to attachment theory, general attachment is developed early through interaction with primary caregivers, whereas specific relationship attachments are developed from experiences within particular relationships (Collins and Read, 1994). During adolescence, attachment figures shift, with peers increasingly taking on a more prominent role (Mitic et al., 2021; Therriault et al., 2024), leading to a divergence between general attachment and parent–child attachment.

The first hypothesis was partially confirmed. Despite being a mediator between attachment avoidance and problem behaviors, parent–child attachment did not significantly mediate the connection between attachment anxiety and problem behaviors. This is consistent with the emotional security hypothesis, which claims that insecure attachment weakens parent–child attachment, resulting in an emotional insecurity among adolescents. Early-formed attachment models strongly influence social behavior patterns (Fraley, 2002), where insecure general attachment (anxious or avoidant) tends to lead to insecurity in parent–child attachment. This insecurity places individuals in a more distressed psychological state, increasing the risk of problem behaviors (Cooke et al., 2019; Jianhua et al., 2025; Wang et al., 2024). Specifically, a person with attachment avoidance usually maintains an emotional distance from his or her child, resulting in a lack of emotional support and security for parent–child relationships. As a consequence, internal insecurity becomes worse and externalizing problem behaviors occur (Mikulincer and Shaver, 2010).

However, parent–child attachment did not significantly mediate the relationship between attachment anxiety and adolescent problem behaviors. One possible explanation is that adolescents with high attachment anxiety tend to exhibit strong emotional dependence on their parents and an ongoing need for reassurance (Mikulincer and Shaver, 2010). This attachment pattern may foster a subjective sense of emotional connection and stability within the parent–child relationship, even when the relationship is characterized by ambivalence or conflict. As a result, the mediating role of parent–child attachment in the link between attachment anxiety and problem behaviors may be weakened. In contrast, attachment avoidance appears to have a more pronounced impact on the quality of the parent–child relationship. Prior research has shown that avoidantly attached individuals often exhibit emotional detachment, interpersonal distance, and unresponsiveness in close relationships—traits that tend to undermine relationship quality more severely than other forms of insecure attachment (Jang et al., 2002; Wang J. et al., 2021; Wang Y. et al., 2021). Consequently, adolescents with high attachment avoidance may be more likely to damage the quality of parent–child attachment, thereby increasing the likelihood of behavioral problems through this disrupted relational pathway.

Moreover, the association between attachment anxiety and internalizing problems such as anxiety and depression has received more consistent empirical support (Barone et al., 2020, 2021; Vernon and Moretti, 2024). In contrast, avoidantly attached adolescents are more likely to engage in externalizing behaviors—such as aggression or rule-breaking—as a means of deflecting attention away from attachment-related distress (Barone et al., 2021). Therefore, the adverse effects of attachment anxiety on adolescent adjustment may be more likely to operate through emotion-related mechanisms—such as emotional dysregulation (Cohen-Bausi et al., 2025), rumination (Seyed Mousavi et al., 2024), and rejection sensitivity (Topino et al., 2025)—rather than parent–child attachment.

We confirmed the second hypothesis. Following the dual-system model of self-control, it is anticipated that attachment anxiety and avoidance will have separate impacts on adolescent problem behaviors. The control system mediates the link between attachment avoidance and problem behaviors, while the impulsive system mediates the connection between attachment anxiety and problem behaviors. When adolescents face challenges in self-control, these two systems compete to predict their behavior, and the dominant system is determined by the relative strength of its activation (Miller et al., 2009). Adolescents with attachment avoidance tend to maintain distance from attachment figures. This strategy depletes cognitive resources and weakens the functioning of the control system (Carmichael and Tyler, 2012), making impulsive behaviors more likely. Adolescents with attachment anxiety, due to a lack of sufficient security from attachment figures, adopt over-activated strategies to cope, making them more prone to impulsivity and a lack of control (Mikulincer and Shaver, 2010), which also increases the risk of problem behaviors. Thus, differences in self-control among adolescents with different general attachment styles further affect their problem behaviors.

The third hypothesis was confirmed to a certain extent. It was found that attachment avoidance and problem behaviors are mediated by a chain of parent–child attachment and control systems. The analysis showed no significant association between attachment anxiety and problem behaviors via the mediating pathways of parent–child attachment and impulsive systems, though the results approached critical significance levels. The internal working model theory posits that securely attached individuals develop more optimistic self-perceptions and social expectations (Bowlby, 1973). A secure internal working model fosters adolescents’ confidence in their parents’ availability and responsiveness as attachment figures, functioning as a secure base, which enables them to have more self-regulation and interpersonal coping resources when facing external pressures and challenges, resulting in emotional stability and positive behavioral outcomes (Mikulincer and Shaver, 2010). In such adolescents, healthy interpersonal relationships are more likely to be formed, emotional control and self-control are stronger (Zimmermann, 1999), enabling them to cope with difficulties and stresses in life more effectively (Jiang et al., 2022). Avoidant attachment, on the other hand, leads adolescents to develop a negative view of others, believing that other people are unable to provide reliable emotional support. Thus, they tend to withdraw emotionally and reduce their engagement in close relationships (Mikulincer and Shaver, 2010). This negative working model of others makes it difficult for them to access the necessary support during distress, hindering their ability to form healthy interpersonal relationships and emotional security (Jiang et al., 2022). Consequently, they may face more significant challenges in managing emotional and behavioral issues.

### Limitations and implications

Several limitations are present. First, it focused solely on the associations between attachment relationships (such as general attachment and parent–child attachment), self-control, and adolescent problem behaviors, without considering other important forms of attachment. According to the hierarchical model of attachment, relationship-specific attachment patterns formed with peers (Flykt et al., 2021; Hay Man, 2022) and teachers (Nulman and Alkalay, 2025; Longobardi et al., 2024) are also important during adolescence and may predict the development of problem behaviors. Future research should expand the scope of attachment assessment to include these specific relational contexts, in order to provide a more comprehensive understanding of how attachment functions across diverse social relationships. Second, problem behaviors were measured only using the self-report version of the Strengths and Difficulties Questionnaire (Kou et al., 2007), which may introduce self-report bias. To enhance the reliability and validity of measurement, future studies are encouraged to include the parent and teacher versions of the SDQ and to adopt a multi-informant approach that gathers data from adolescents, parents, and teachers (Kou et al., 2005). Finally, this study used a cross-sectional design, which limits the ability to conclude the directionality of the observed associations. Future research could adopt an attachment priming paradigm to observe changes in self-control under attachment-activated conditions, as well as adolescents’ behavioral responses in specific scenarios. For example, research has shown that both trait-based and situationally activated attachment security can enhance response inhibition following ego-depletion (Li et al., 2016). Additionally, another study found that under ego-depletion conditions, priming attachment security improved support-providing behaviors, which are regulated by executive functioning (Mikulincer et al., 2013).

Despite the above-mentioned limitations, this study has important theoretical and practical implications. First, within the framework of the family ecological systems theory, this research integrates the attachment hierarchy model with the dual-system theory of self-control, offering a novel and systematic perspective on how general attachment predicts adolescent problem behaviors through the mediating roles of parent–child attachment and self-control. The study further reveals that different types of insecure attachment predict adolescent problem behaviors through distinct self-control systems: Attachment anxiety was associated with heightened impulsive tendencies, whereas attachment avoidance was linked to weaker control system functioning. These findings suggest that the two dimensions of insecure attachment may be differentially related to the dual systems of self-control in the context of adolescent problem behaviors. Second, by including both general attachment and parent–child attachment, the study reveals the independent and joint effects of different levels of attachment in predicting adolescent problem behaviors.

On a practical level, the findings offer clear guidance for intervention strategies. On one hand, enhancing adolescents’ attachment security and caregiving quality can promote the improvement of parent–child attachment and self-regulation abilities. For example, attachment-based parenting interventions (such as the Connect program) have been shown to effectively reduce adolescent problem behaviors (Barone et al., 2021; Benzi et al., 2023) On the other hand, interventions should be tailored to different attachment styles: for adolescents with avoidant attachment, the focus should be on restoring emotional connections with parents to activate their control systems; for those with anxious attachment, emphasis should be placed on improving emotional regulation and reducing maladaptive coping strategies such as hypervigilance and rumination. In addition, schools may consider designing self-control training programs that take adolescents’ attachment profiles into account, allowing for more tailored and developmentally sensitive support.

## Conclusion

In conclusion, the principal results as follows: First, parent–child attachment mediates between attachment avoidance and behavioral problems. Second, the mechanisms of the two self-control systems differ in predicting adolescent behavioral problems; the control system mediates between attachment avoidance and behavioral problems; the impulsive system mediates between attachment anxiety and behavioral problems. Finally, parent–child attachment and the control system mediate between attachment avoidance and behavioral problems.

## Data Availability

The raw data supporting the conclusions of this article will be made available by the authors, without undue reservation.
